# Exploring Surgical Management Strategies for Endobronchial Tumors

**DOI:** 10.7759/cureus.73036

**Published:** 2024-11-05

**Authors:** Chau P Thi, Vu Huu Vinh, Lam X Nhat

**Affiliations:** 1 Department of Thoracic Surgery, Cho Ray Hospital, Ho Chi Minh, VNM

**Keywords:** diagnosis, endobronchial tumors, lobectomy, surgical treatment, video-assisted thoracic surgery

## Abstract

Endobronchial tumors, though relatively uncommon, present a diverse array of pathological conditions, including both benign and malignant neoplasms. This retrospective study focuses on the surgical management of seven such cases affecting the main or lobar bronchi. The study involves a diverse patient population, ranging in age from 18 to 65 years, with a median age of 26 years. The cases include both male and female individuals, indicating the presence of these tumors across various demographics. We carried out surgical interventions to remove the tumor while preserving the lung parenchyma. Surgical techniques included video-assisted thoracic surgery (VATS), lobectomy, and/or a combination of these methods. The choice of approach was guided by the tumor's location and nature. VATS, although widely performed, can sometimes pose challenges, leading to conversions to open thoracotomy in specific cases. All seven cases resulted in uneventful postoperative periods and excellent long-term outcomes, affirming the efficacy of the surgical procedures. Notably, despite variations in age and gender, the treatment approaches consistently yielded favorable long-term results. This study demonstrates the importance of personalized surgical strategies for endobronchial tumors while considering the nature and location of the tumor. VATS stands as a viable option, particularly in benign cases, provided careful case selection and the availability of skilled surgeons. These findings contribute to the growing body of evidence supporting the benefits of VATS in the management of endobronchial tumors.

## Introduction

Endobronchial tumors affecting the main or lobar bronchi are relatively uncommon [1]. Such tumors can be benign, carcinoid (a neuroendocrine tumor with a good prognosis), or malignant (carcinoma). Endobronchial tumors cause atelectasis due to obstruction of the bronchi or abscess in the distant lobes of the lung [2]. In many patients, diagnosis is wrongly made for lung tumors. Histopathological establishment of the nature of the tumors is essential for determining the treatment strategy (as removal of tumor only versus sleeve bronchial reconstruction or lobectomy). Endobronchial neoplasms have diverse pathological presentations, with malignant tumors being more common than benign tumors. Surgical removal of the tumor is preferred in most cases, but complex bronchial reconstruction procedures are attempted if expertise is available, especially in patients with compromised cardiopulmonary reserve status. Although video-assisted thoracic surgery (VATS) is a viable option for all the above-mentioned surgeries, its success is subject to the surgeon’s case selection, expertise, and experience [3]. Surgical removal of the tumor with parenchymal sparing is considered a tough clinical challenge, and it is preferred in most cases of endobronchial neoplasms [4-6]. However, in cases of malignant endobronchial tumors, a routine segmental bronchial resection is insufficient for achieving a tumor-free margin, usually in metastatic lymph node patients [4,7]. Herein we have documented seven cases of endobronchial tumors where VATS was done with or without lobectomy. Of the 7 cases, 4 were benign and 3 were malignant. Among the malignant cases, one was squamous cell carcinoma, one was low-grade mucoepidermoid carcinoma, and one was carcinoid. Although the outcomes were satisfactory and there was no mortality, it is to be noted that the surgeon’s case selection, expertise, and experience are the essential factors to be considered before deciding on VATS. Preoperative bronchoscopy-induced bleeding can be challenging, as seen in two cases. Instead, frozen section biopsy can be done during the VATS procedure much more safely.

## Materials and methods

This retrospective study aimed to investigate the surgical management of endobronchial tumors. It was conducted at Choray Hospital in Vietnam over three years (2020-2022). All patients admitted to the hospital during this period with tumor resection and bronchial reconstruction, lobectomy, or sleeve lobectomy were considered. The study design involved the analysis of diagnostic considerations, surgical approaches, and outcomes. Data collection involved the compilation of several pieces of information for each case. These included demographic information, including the age and gender of the patient, and details regarding the location of the endobronchial tumor. Seven cases, four females and three males, who presented with endobronchial tumors affecting the main or lobar bronchi were included in this study. The age of the participants varied from 18 to 65 years. The affected bronchial segments included the right main bronchus in two female patients (26 and 20 years old), the right intermediate bronchus in a 65-year-old female and a 40-year-old male, and the right superior bronchus in an 18-year-old male. Additionally, a 27-year-old female presents with pathology in the left inferior bronchus, while a 62-year-old male demonstrates issues in the left superior bronchus. Surgical procedures were documented to report whether video-assisted thoracic surgery (VATS), lobectomy, or a combination of these methods were employed. Additionally, the outcomes of the procedures, which included evaluations of the postoperative period and long-term results, were scrutinized. Histopathological examinations were also performed on resected tumor specimens to establish the nature of the tumors and confirm the diagnosis. Descriptive statistics were employed to provide a comprehensive summary of the data collected from the cases. The research was conducted in strict adherence to ethical guidelines, and informed consent was obtained from the patients to ensure their willingness for the publication of their medical information.

## Results

This study presents the surgical management of seven cases of endobronchial tumors, both benign and malignant. The average age of the patients was approximately 38.57 years, with a relatively wide age range spanning from 18 to 65 years. The median age was 26 years. This data indicates the diversity in age among the individuals affected by bronchial tumors. Regarding gender distribution, four out of the seven cases involved female individuals, while the remaining three cases pertained to male patients. In terms of patient outcomes, all seven cases reported successful results. Each patient underwent surgical interventions that led to uneventful postoperative periods with excellent long-term outcomes. This uniform positive outcome demonstrates the effectiveness of the surgical procedures applied in these cases, as well as the skill and expertise of the healthcare teams involved. It is noteworthy that despite the diversity in patient age and gender, the treatment approaches consistently yielded favorable long-term results. A summary of the cases is presented in Table 1.

**Table 1:** Summary of the cases.

Age	Gender	Tumor Location	Surgical Procedure	Outcome	Diagnosis
26	Female	Right main bronchus	VATS with pulmonary sleeve resection and reconstruction of the main bronchus	Uneventful post-op, excellent long-term	Squamous cell carcinoma
20	Female	Right main bronchus	VATS Bronchial sleeve tumor resection	Uneventful post-op, excellent long-term	Low-grade mucoepidermoid carcinoma
65	Female	Right intermediate bronchus	Open thoracotomy with right upper lobe removal	Uneventful post-op, excellent long-term	Myoepithelioma
40	Male	Right intermediate bronchus	VATS bilobectomy (R middle and lower) with middle and lower lobectomies, lower lobectomy of the right lung	Uneventful post-op, satisfactory outcome	Carcinoid tumor
18	Male	Right superior bronchus	Open thoracotomy with right upper lobe removal	Satisfactory outcome	Mucous gland adenoma
27	Female	Left inferior bronchus	VATS inferior bronchial tumor resection	Excellent outcome	Mucous gland inflammation
62	Male	Left superior bronchus	VATS converted to thoracotomy with upper lobectomy of left lung	Uneventful post-op, excellent long-term	Mucous gland inflammation with tuberculosis infection

In the first case, a 26-year-old female patient was diagnosed with a lung tumor in the right main bronchus. The surgical approach involved VATS with pulmonary sleeve resection and primary bronchial reconstruction. All three lobes were successfully preserved, and the post-operative course resulted in excellent long-term outcomes. The histopathological examination confirmed squamous cell carcinoma. The second case involved a 20-year-old female patient with a tumor in the right main bronchus, initially referred from another hospital. CT scans revealed bronchial obstruction, while bronchoscopy confirmed the diagnosis. VATS was employed to remove the tumor, avoiding the need for lobectomy. The patient's postoperative recovery was uneventful, leading to excellent long-term results. Histopathological examination identified the tumor as low-grade mucoepidermoid carcinoma. In the third case, a 65-year-old female patient underwent bronchoscopy for the diagnosis of a tumor in the right intermediate bronchus. Unfortunately, a severe hemorrhage occurred during the biopsy, necessitating immediate ECMO support. Emergency surgery, beginning with VATS and transitioning to open thoracotomy with right upper lobe removal (lobectomy), was performed to manage bleeding from an injured branch of the right pulmonary artery. The patient experienced an uneventful postoperative period with an excellent long-term outcome. Histopathological examination revealed myoepithelioma. The fourth case involved a 40-year-old male patient with a tumor in the right intermediate bronchus, diagnosed through CT scans and bronchoscopy. The preoperative bronchoscopy biopsy confirmed a typical carcinoid tumor. VATS was used for tumor removal, which included bilobectomy of the middle and lower lobes of the right lung due to the tumor's location and histopathological diagnosis. The patient's postoperative period was uneventful, signifying a satisfactory outcome. In the fifth case, an 18-year-old male presented with a tumor in the right superior bronchus. Despite successful VATS tumor removal, an accidental injury to the right pulmonary artery led to excessive hemorrhage. Consequently, an open thoracotomy and right upper lobe excision (lobectomy) were necessary to control the bleeding. The tumor's size and location necessitated lobectomy. The outcome was satisfactory, and histopathological examination confirmed the diagnosis of mucous gland adenoma. In the sixth case, a female patient, 27 years old, had a tumor detected in the left inferior bronchus by bronchoscopy. VATS was successfully employed without the need for lobectomy. Histopathological examination revealed inflammation of the mucous gland, and the outcome was excellent. Finally, in the seventh case, a 62-year-old male patient presented with a tumor in the left superior bronchus, which was confirmed through bronchoscopy and CT scans. Unfortunately, a bronchoscopy biopsy resulted in massive bleeding necessitating ECMO support. This was followed by an open thoracotomy and upper lobectomy of the left lung to manage excessive hemorrhage due to accidental injury to the pulmonary artery. Histopathological findings revealed both mucous gland inflammation and tuberculosis infection. The patient's postoperative period was uneventful, leading to an excellent long-term outcome.

## Discussion

Sleeve lobe resection and carina reconstruction are sometimes performed on malignant endobronchial tumors [8,9]. In comparison to malignant cases, in patients having benign or low-grade malignant endobronchial tumors, complex bronchial reconstruction procedures are attempted if expertise is available [4,10]. In these cases, the surgeon(s) tried to preserve normal lung tissue to the most feasible extent, especially in patients with compromised cardiopulmonary reserve status. In both benign and malignant endobronchial tumor patients, lobectomy can be done [11-13]. VATS is widely performed due to its association with lower incidences of postoperative complications, more tolerable postoperative pain, and earlier pulmonary function recovery compared to thoracotomy [4,11].

However, there is still room for debate on whether VATS is recommended in benign endobronchial tumors. However, some cases might lead to complications like excessive bleeding during VATS and needing conversion of VATS to thoracotomy [4,11]. In a retrospective study by Kim D and his associates, the effectiveness and practicality of VATS lobectomy were assessed in benign pulmonary diseases [11]. They found that 163 patients (42%) underwent VATS lobectomy; among all these patients, in only six patients (4%), VATS lobectomy was converted to thoracotomy. They also found no statistical differences in blood loss and operation time during VATS lobectomy in the patients with or without infection. Similar to this study, our study focused on bronchial tumors rather than lung tumors. The goal of our procedure was to resect the tumor without performing a lobectomy, if possible. The VATS approach can be more challenging in bronchial sleeve reconstruction compared to lobectomy. In our seven cases, two (28%) required conversion to open surgery due to massive bleeding resulting from accidental pulmonary artery injury caused by prior bronchoscopy procedures.

Although compared to malignant cases, in benign pulmonary diseases, the incidence of associated infection is relatively high. Hence, some surgeons prefer open thoracotomy compared to VATS. In the study by Kim D and his colleagues mentioned above, VATS’s viability, rationality, and effectiveness have been well established compared to open thoracotomy [11]. Also based on the low conversion rate from VATS to open thoracotomy and excellent long-term outcome reinforced VATS’s usefulness in managing benign endobronchial diseases. Findings from our study also support the same recommendation. However, special consideration should be given while selecting the cases. In the study published by Kim D, patients with pleural adhesion detected on preoperative CT scans, especially in the hilar region, were not selected for VATS. Instead, they underwent thoracotomy [11]. Moreover, like any other minimally invasive surgical procedure, VATS requires experienced and skilled surgeons who might not be available in all hospital settings. Hence, despite being the most recommended procedure for benign endobronchial tumor management, case selection and availability of skilled and experienced surgeons are essential for performing VATS successfully.

The lack of randomized clinical trial data comparing morbidity and mortality following lobectomy done by open thoracotomy and VATS led Paul S and his associates to conduct a propensity-matched analytical study based on the Society of Thoracic Surgeons (STS) database [14]. The researchers found that compared to open thoracotomies, VATS’s lobectomies show lower postoperative complication rates besides lower individual complication rates, including atrial fibrillation. Although such study findings do not amount to level 1 evidence of the randomized clinical trial results, they effectively addressed the problem of biases associated with retrospective single-institution case series [14]. It has been demonstrated by several institutions that fewer postoperative complications may be associated with video-assisted thoracoscopic lobectomy compared to open thoracotomy [14-17]. Despite all the extraordinary evidence, the application of VATS, in reality, remains far more limited. The lack of experienced and skilled surgeons comfortable with performing VATS is one of the main factors for less application of VATS. Often, surgeons do not get to learn the procedure (VATS) during their training periods. Also, there are concerns among them regarding the procedure’s efficacy in managing malignant cases.

However, studies have shown that if taught and practiced by a confident and skilled surgeon, the procedure is at least as effective as open thoracotomy in malignant cases. Again, the idea that lobectomies performed using VATS might have lesser postoperative complication rates has been explored in a single institution study performed by Park BJ and his colleagues [18]. Although the postoperative complications’ overall incidence was significantly lower (p=0.046) in patients undergoing VATS lobectomy, compared to age and sex-matched patients undergoing open thoracotomy lobectomy, no significant differences were observed in terms of specific complications. Moreover, incidences of atrial fibrillation as a postoperative complication were similar in patients undergoing VATS compared to those who underwent lobectomy using open thoracotomy.

In another study when the incidence of postoperative complications of VATS compared to open thoracotomy was analyzed in elderly patients, it was found that VATS was significantly safer (p=0.04) in those patients compared to open thoracotomy [18]. Cattaneo SM and his colleagues, in another study, assessed if lobectomy performed in elderly patients via VATS for clinical stage I non-small cell lung cancer subsides complications compared to lobectomy performed through open thoracotomy (THOR) [19]. They found that in stage I non-small cell lung cancer in elderly patients, the VATS approach is rather advantageous, as in their study, with a significantly lesser number of postoperative and perioperative complications and significantly shorter hospital stays compared to patients who underwent lobectomy by THOR [19]. In another retrospective study, Kim and his associates reviewed the short-term and midterm outcomes following VATS thoracotomy, considering factors such as postoperative morbidity, recurrence, mortality, and survival of early-stage non-small cell lung cancer (NSCLC) patients who underwent lobectomy by VATS [20]. VATS was considered a technically feasible and rational operative recourse for the early stage of NSCLC because of its association with a lesser incidence of complications, a shorter duration of hospital stay, and being minimally invasive [20]. Although only benign endobronchial cases were included in our study, we also found that the feasibility, safety, and shorter hospital stay associated with VATS reinforce the utility and advantage of VATS in endobronchial tumor surgery with or without lobectomy.

A critical factor influencing the surgical strategy is the tumor's histopathological nature and location. For instance, in our study, the presence of squamous cell carcinoma in a younger patient and the identification of a carcinoid tumor in another necessitated more aggressive surgical interventions, including sleeve resection and bilobectomy. These tumor characteristics often dictate the extent of resection required to achieve clear margins while preserving lung function.

The conversion of VATS to open thoracotomy in two cases was necessitated by intraoperative challenges, such as excessive bleeding. These conversions underscore the limitations of VATS in certain scenarios, particularly when unexpected complications arise. For example, in one case, a severe hemorrhage occurred during bronchoscopy, necessitating immediate ECMO support and conversion to thoracotomy. In another instance, VATS was initially attempted but was converted to thoracotomy due to complications with tumor resection. These cases emphasize the importance of meticulous preoperative planning, including consideration of factors like pleural adhesion and the tumor's proximity to major vessels, which may not always be fully appreciated until intraoperative exploration.

These findings suggest that while VATS offers significant advantages, including reduced postoperative pain and quicker recovery, its application must be carefully weighed against potential risks, especially in complex cases. The decision to convert to open thoracotomy should not be viewed as a failure but rather as a necessary adjustment to ensure patient safety and optimal outcomes.

## Conclusions

In patients with localized malignancy and those unwilling to undergo surgical intervention, radiation therapy, especially brachytherapy, is advised. Lobectomy can also be implemented in both benign and malignant endobronchial tumor patients. VATS is widely executed and associated with lower incidences of postoperative complications, more tolerable postoperative pain, and earlier pulmonary function recovery compared to thoracotomy. However, there is still debate on whether VATS is recommended in benign endobronchial tumors due to the availability of fewer studies comparing the advantages of VATS and open thoracotomy. Recent studies have shown that VATS lobectomy remains an effective and practical choice in benign pulmonary disease, irrespective of infection status, but special consideration should be given while selecting the cases and the need for experienced and skilled surgeons.
